# Optimising medication data collection in a large-scale clinical trial

**DOI:** 10.1371/journal.pone.0226868

**Published:** 2019-12-27

**Authors:** Jessica E. Lockery, Jason Rigby, Taya A. Collyer, Ashley C. Stewart, Robyn L. Woods, John J. McNeil, Christopher M. Reid, Michael E. Ernst, on behalf of the ASPREE Investigator Group

**Affiliations:** 1 Department of Epidemiology & Preventive Medicine, Monash University, Melbourne, Victoria, Australia; 2 School of Public Health, Curtin University, Perth, Western Australia, Australia; 3 Department of Pharmacy Practice and Science, College of Pharmacy and Department of Family Medicine, Carver College of Medicine, The University of Iowa, Iowa City, Iowa, United States of America; Victoria University, AUSTRALIA

## Abstract

**Objective:**

Pharmaceuticals play an important role in clinical care. However, in community-based research, medication data are commonly collected as unstructured free-text, which is prohibitively expensive to code for large-scale studies. The ASPirin in Reducing Events in the Elderly (ASPREE) study developed a two-pronged framework to collect structured medication data for 19,114 individuals. ASPREE provides an opportunity to determine whether medication data can be cost-effectively collected and coded, *en masse* from the community using this framework.

**Methods:**

The ASPREE framework of type-to-search box with automated coding and linked free text entry was compared to traditional method of free-text only collection and *post hoc* coding. Reported medications were classified according to their method of collection and analysed by Anatomical Therapeutic Chemical (ATC) group. Relative cost of collecting medications was determined by calculating the time required for database set up and medication coding.

**Results:**

Overall, 122,910 participant structured medication reports were entered using the type-to-search box and 5,983 were entered as free-text. Free-text data contributed 211 unique medications not present in the type-to-search box. Spelling errors and unnecessary provision of additional information were among the top reasons why medications were reported as free-text. The cost per medication using the ASPREE method was approximately USD $0.03 compared with USD $0.20 per medication for the traditional method.

**Conclusion:**

Implementation of this two-pronged framework is a cost-effective alternative to free-text only data collection in community-based research. Higher initial set-up costs of this combined method are justified by long term cost effectiveness and the scientific potential for analysis and discovery gained through collection of detailed, structured medication data.

## Background

Pharmaceuticals play an important role in clinical care and, consequently, there is a need to optimise collection of concomitant medication in clinical research. In the past, data collection on paper has dominated clinical research [1], however the digital age presents significant opportunity for efficiency and improvements to data quality. Optimal workflows for collecting coded concomitant medication data in an accurate, cost-effective manner are needed for large, community-based studies [2] because concomitant medication exposure is important, but the collection, coding and analysis of these data can be challenging. Traditionally, clinical trial medication data are collected as unstructured text (on paper or electronically) by data collectors without specialised knowledge of medications. This text is later coded manually by a pharmacist or clinician to produce a structured, coded data set for analysis [2,3]. Smaller studies can afford to manually review and code electronic medication data [4] but this process can be prohibitively expensive for large-scale clinical trials.

Medication data collected for the purpose of clinical care within the hospital, outpatient or community health centre environment is generally entered directly into the Electronic Health Record (EHR) using complex, pre-specified data entry fields or Computerized Physician Order Entry (CPOE) systems. Researchers within these environments can leverage EHRs and CPOE systems to gather medication data [5]. Additionally, EHR coding tools (*e*.*g*. RxNorm) can be integrated within a research user interface allowing users to select medications from a pre-configured dropdown box [6]. However, chronic medication use extends outside the hospital setting and many important clinical insights are gained via community-based research. In contrast to hospital based research where there is usually a high level of familiarity with medications and medication data, community-based research is often conducted outside the hospital data infrastructure using data collectors who are not clinically trained. This makes collection of vital medication data particularly challenging in community based research. It is unknown whether frameworks shown to be effective for hospital-based research, such as a pre-configured medication list, are appropriate for community-based data collectors without specialised medication knowledge. There is also no empirically-derived estimate of the potential cost savings associated with the implementation of an framework for structured medication data collection in a community-based research setting.

ASPirin in Reducing Events in the Elderly (ASPREE) was a randomised, placebo controlled clinical trial of aspirin in 19,114 community-dwelling older people in Australia and the US [7,8]. Given that prescription medication use is prevalent in the elderly and has the potential to impact clinical outcomes [9–12], ASPREE collected annual prescription medication data for the duration of the study because of the relevance of medication to understanding of health issues affecting an elderly population. Data collection in ASPREE was supported by the ASPREE Web Accessible Relational Database (*AWARD*) suite, which facilitated collection of medication data in structured format or as free-text collection. ASPREE provides the opportunity to assess the utility of a pre-configured *structured* medication data collection tool in a research setting where data collectors are not pharmacy or medically trained. In this paper we analyse the utility of this framework by comparing entry of medication in a structured format with free-text entries, assessing factors contributing to the entry of free-text data entry, and suggesting how medication data collection can be optimised in the setting of a large-scale community-based clinical trial.

## Methods

### ASPREE clinical trial

In this analysis data were examined from randomised ASPREE participants. Briefly, ASPREE was a randomised placebo-controlled trial of low-dose (100mg) aspirin in 19,114 healthy people aged 70 years or older (65 or older for US minorities) without previous cardiovascular disease, conducted in Australia (n = 16,703) and the USA (n = 2,411) [13]. The study commenced March 2010 and concluded in June 2017, with a median 4.7 years of follow-up including annual data collection. At baseline, ASPREE participants were required to be in good health, free of major diseases and expected to survive 5 years of follow-up (confirmed by the participant’s general practitioner/primary care provider). ASPREE was approved by multiple Institutional Review Boards in Australia and the US prior to data collection, and all the participants provided written informed consent for data collection. Detailed methods and results of ASPREE are described in detail elsewhere [7,13].

### Collecting structured medication data

**Fig 1:**
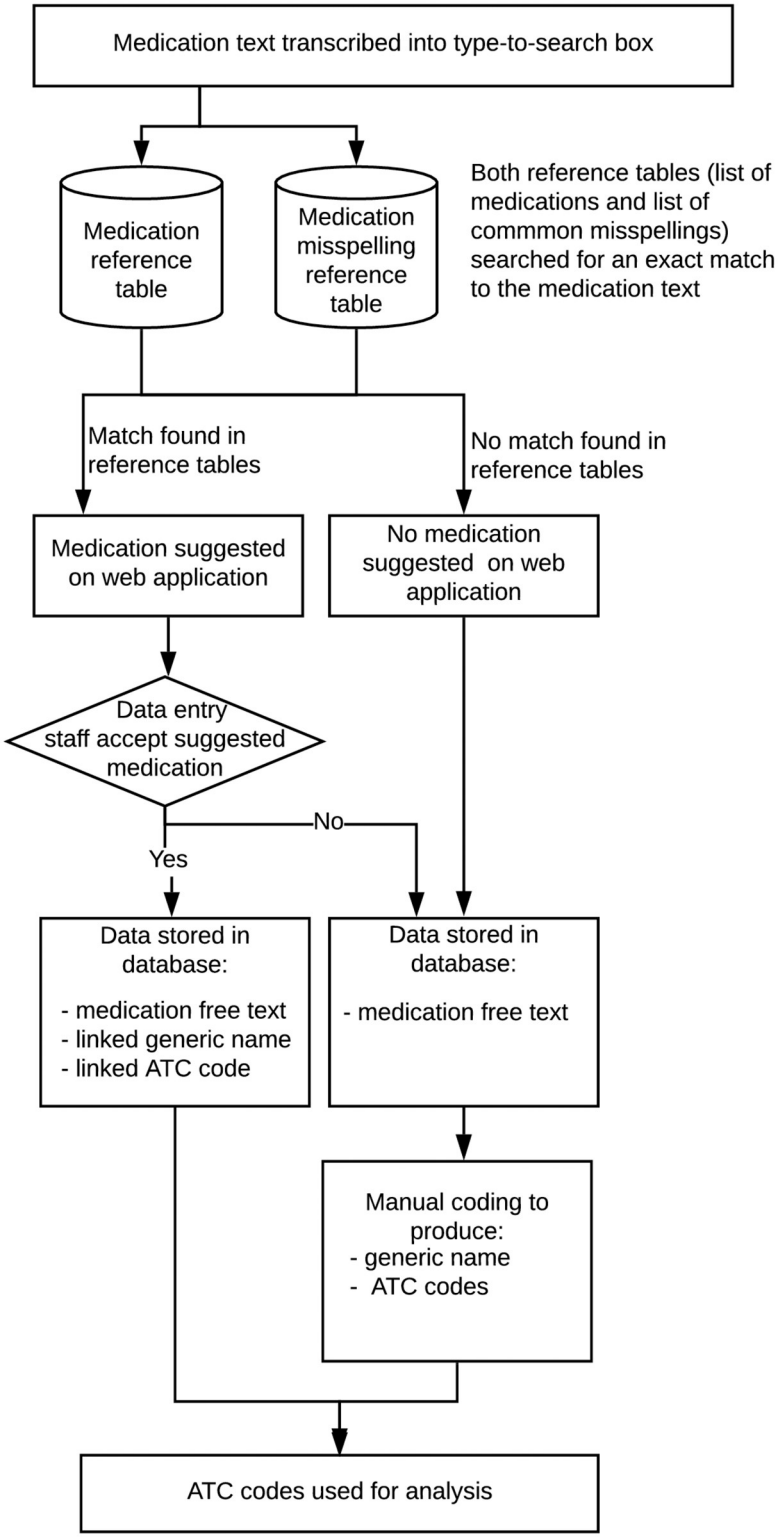
ASPREE concomitant medication data collection framework.

The ASPREE Web Accessible Relational Database (*AWARD)* suite facilitated electronic collection and storage of medication data via a two-pronged framework (See Fig 1). There was a concern that staff would become confused if they were presented with an overwhelming number of medication options on the electronic medication data collection form in *AWARD*. Therefore, a list of 2025 common medications, which included generic and/or trade names, was compiled at the commencement of the study. This list was based on free-text medication data collected in the pilot phase of ASPREE and coded medication data from other community-based studies conducted by Monash University. This list of common medications was made available to users as a type-to-search text box on the electronic version of the medication case report form in *AWARD*. Staff entered structured medication data via the type-to-search box or, if the medication name was not found, selected ‘Other’ and entered the reported medication as free-text. To support entry of medications using the type-to-search box a mapping table in the *AWARD* database provided a link between common medications misspellings and the generic name. If a common misspelling was entered, the type-to-search box offered the linked generic name, which allowed the data entry staff to select either the generic name or enter the misspelling as free-text (see Fig 1). The list of common medications was not updated during the course of the study.

### Collection of medication from participants

ASPREE participants were asked to bring their medications, or an updated medication list, to their baseline data collection visit and every annual visit thereafter. Research staff reviewed each medication and confirmed whether the medication was prescribed by the participant’s doctor. The names of all prescription medications (preferably generic) and the participant self-reported medication commencement and cessation year were transcribed onto a case report form (CRF). Dose information was not within the scope of data collection for ASPREE. However, participants incidentally and voluntarily reported dose for certain medications. In this situation some staff chose to enter medication data as free text so that the dose was recorded in the database. In addition to all prescription medications, other medications of relevance to the aspirin intervention and main outcomes in ASPREE were recorded if participants reported taking them at least once per week on a regular basis, regardless of whether they were prescribed by a doctor or obtained without prescription. These included nonsteroidal anti-inflammatory drugs, paracetamol/acetaminophen, vitamin D and open label aspirin. When available, staff utilised clinic medical records to prompt participants about medications that they may have forgotten, with the aim of producing a comprehensive list. The majority of data collection staff did not have specialised medication knowledge but received standardized training at study onset in how to identify medication names from packaging and how to complete data entry using the type-to-search box.

### Medication coding and validation

All medications were linked to their generic name and coded according to the World Health Organisation Anatomical Therapeutic Chemical (ATC) codes. For free-text medications, a Neural Network was utilised to produce a ranked list of 20 potential ATC codes for each free-text entry. This list was delivered to two independent coders via a web application (see S1 Fig). Coders selected the correct ATC code from the list or entered a new code if the correct result did not appear in the ranked list. To accommodate reports of combination medications or simply multiple medications being batched together, the code allowed for each free text report to be linked to multiple ATC codes. This allowed the coder the flexibility to select as many ATC codes as were necessary based on the active ingredients listed in medication report text. Discordance between coders was resolved by a third expert coder. Combination medication entries were split into component ingredients prior to coding, and each assigned the appropriate ATC code. Approximately half of the coders were clinically trained (*i*.*e*. medical practitioners or pharmacists). The coder included links to Google, the Australian National Prescriber Service website, and the World Health Organisation Anatomical and Therapeutic Chemical (ATC) coding website to support non-clinically trained coders to access the information required to correctly identify a drug and select the appropriate ATC code (see S1 Fig).

### Costing

Relative cost of medication collection was determined based on the time required for database programming and medication coding. Database programming was estimated based on the average time required to set up, restrict and deploy an SQL table and linked web application data entry page, multiplied by the number of tables requiring configuration. To determine time required to code each medication, three coders completed a series of coding ‘time trials’. During the time trial coders were randomly delivered different medications by the coder, which they then coded. The time taken to complete coding was dependent upon the complexity of the report (*e*.*g*. combination medication or obscure medication vs clear non-prescription medication or common medication) and the experience of the coder. Each time trial spanned 15 minutes and each coder completed 4 trials. The median time to code a medication was then calculated and used for determination of cost.

## Results

**Fig 2:**
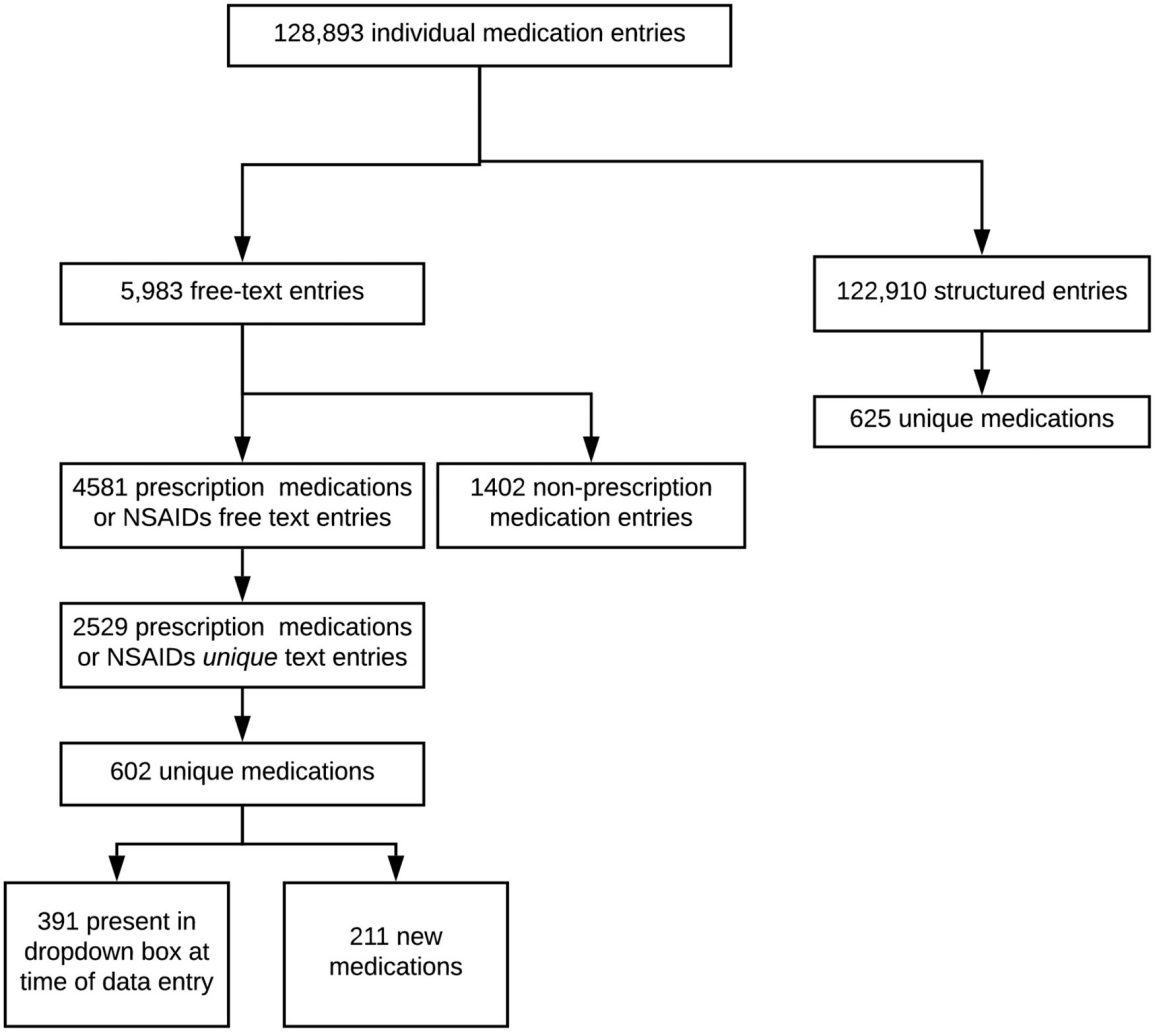
Results of medication collection framework for ASPREE trial.

As shown in Fig 2, 128,893 unique participant medication reports were entered into *AWARD-*Data. Of these, 122,910 (95%) were captured within a structured variable using the type-to-search box, and 5983 (5%) were entered as free-text. Of the free-text reports, 4,581 were confirmed to be prescription medications or NSAIDs, and 1,402 were confirmed to be non-prescription medications (*e*.*g*. vitamins and complementary medications). For approximately one out of six medication reports, the codes assigned by the first two coders were discordant and a third coder review was required. Overall, free-text data contributed 602 unique medications (*i*.*e*. unique ATC codes), including 211 medications not present in the type-to-search box.

The top ten medications reported via structured and free-text data are shown in Table 1. Paracetamol/acetaminophen was the medication most commonly reported via the type-to-search box (n = 6899), followed by Vitamin D (n = 3582). Cardiovascular drugs such as statins (Atorvastatin, n = 3192; Rosuvastatin, n = 2514; Simvastatin, n = 2000), nicardipine (n = 2307), perindopril (n = 2,303) and open label aspirin (n = 2,542) accounted for six of the ten most commonly reported medications. With regard to medications entered as free-text, the most commonly reported medication was apixaban (n = 600). This medication, along with Tapentadol (n = 88), Aclidinium bromide (n = 72), Umclidinium bromide (n = 71), empaglifozin (n = 50) and dapagliflozin (n = 49) were approved for use in Australia after the type-to-search box was activated and hence not available in the list. Vitamin D (n = 93) and paracetamol/acetominophen (n = 65) were the only medications to appear in both the top ten structured data and free text lists.

**Table 1:** Top ten most commonly reported non-combination medications entered via type-to-search and free-text.

	Entered via type-to-search box			Entered as free-text			
	ATC	Generic Name	N (reports)	ATC	Generic Name	N (reports)	Available in type-to-search box?
1	N02BE01	Paracetamol	6899	B01AF02	Apixaban	600	No
2	A11CC	Vitamin D	3582	A11CC05	Vitamin D^a^	93	Yes
3	A02BC05	Esomeprazole	3500	N02AX06	Tapentadol	88	No
4	C10AA05	Atorvastatin	3192	R03BB05	Aclidinium bromide	72	No
5	B01AC06	Aspirin	2542	R03BB07	Umeclidinium bromide	71	No
6	C10AA07	Rosuvastatin	2514	N02BE01	Paracetamol	65	Yes
7	C08CA01	Nicardipine	2307	A10BK03	Empaglifozin	50	No
8	C09AA04	Perindopril	2303	C01DA02	Glyceryl Trinitrate	50	Yes
9	M01AC06	Meloxicam	2034	A10BK01	Dapagliflozin	49	No
10	C10AA01	Simvastatin	2000	B03BA03	Vitamin B12^b^ (injection)	42	Yes

^a^ As cholecalciferol

^b^ As hydroxocobalamin

As ASPREE data was collected in Australia and the USA, an opportunity arises to explore between-country differences in medication data entry patterns. As shown in Table 2, Australian participants contributed 83.9% of total medication reports, which is broadly consistent with the proportion of Australian participants overall (87% of total cohort). Of these, 95.7% were entered via the type-to-search box compared with 93.2% of reports in the US. Medications from ATC categories for blood, cancer and ‘various’ had the lowest proportion of medications entered as structured data regardless of country of origin (85.2%, 84.2%, and 73.3% respectively). Reasons for entry of medication as free-text are shown in S1 Table. Relevant medication not being available in the type-to-search box was the most common reason for entry of free-text data (44.6% of all free-text) followed by spelling errors (18.9%), provision of additional information such as dose, route or timing of medication (13.7%), and entry of trade name (10.7%). Of the free-text reports of open label aspirin, 73% were due to the provision of additional information regarding dose (n = 22 of 30).

**Table 2:** Method of medication report by country and ATC group.

	AUS		US	
Medication Class	Medications entered as structured data N (% total in class per country)	Medications entered as free-text N(%)	Medications entered as structured data N(%)	Medications entered as free-text N(%)
A - Alimentary Tract And Metabolism	14,660 (97.9%)	319 (2.1%)	2,220 (94.9%)	119 (5.1%)
B - Blood And Blood Forming Organs	4,203 (84.5%)	770 (15.5%)	758 (89.0%)	94 (11.0%)
C - Cardiovascular System	28,234 (97.4%)	767 (2.6%)	4,985 (96.1%)	203 (3.9%)
D - Dermatologicals	2,249 (93.6%)	155 (6.4%)	170 (80.2%)	42 (19.8%)
G - Genito Urinary System And Sex Hormones	3,139 (97.5%)	82 (2.5%)	766 (94.6%)	44 (5.4%)
H - Systemic Hormonal Preparations, Excl. Sex Hormones And Insulins	3,007 (98.8%)	36 (1.2%)	594 (97.5%)	15 (2.5%)
J - Antiinfectives For Systemic Use	2,043 (93.9%)	133 (6.1%)	317 (89.8%)	35 (9.9%)
L - Antineoplastic And Immunomodulating Agents	1,164 (85.5%)	198 (14.5%)	128 (74.4%)	44 (25.6%)
M - Musculo-Skeletal System	8,780 (98.6%)	123 (1.4%)	1,206 (95.3%)	59 (4.7%)
N - Nervous System	18,418 (97.1%)	541 (2.9%)	1,702 (95.3%)	81 (4.5%)
P - Antiparasitic Products, Insecticides And Repellents	336 (94.1%)	21 (5.9%)	33 (86.8%)	5 (13.2%)
R - Respiratory System	4,957 (92.4%)	409 (7.6%)	967 (94.0%)	62 (6.0%)
S - Sensory Organs	1,932 (93.1%)	143 (6.9%)	439 (89.0%)	54 (11.0%)
V - Various	73 (73.7%)	26 (26.3%)	1 (50.0%)	1 (50.0%)
N/A – Non-prescription medication	5449 (83.9%)	1044 (16.1%)	1292 (78.3%)	358 (13.5%)
N/A - Combinations	7823 (95.5%)	365 (4.5%)	1033 (92.7%)	81 (7.3%)
TOTAL	106,330 (95.7%)	4767(4.3%)	16,580 (93.2%)	1216 (6.8%)

### Relative cost of medication collection and coding

The results of the coding time trials are shown in S2 Table. Overall, 588 medication reports were coded as part of the time trials, which was 9.8% of all the free-text reports. The median time to code a single medication was 18.7 seconds (IQR = 22). Given that each free-text report required coding by two coders (see Fig 1) and one out of six were discordant and required review by a third coder, a total time of 40.5 seconds was used for calculation of the overall cost of coding (18.7*2 + 18.7/6). Detailed calculation of cost per method of collection is shown in Table 3. Collection of medication data using the *AWARD* system required 113.8 hours of funded support for setup (*i*.*e*. database and web application development, generation and coding of options in type-to-search box) and 28.5 hours of funded support for *post hoc* coding of free-text medication reports. Overall, the cost per medication using this framework was estimated to be USD $0.03. Implementation of free-text data collection only, without support of the type-to-search functionality, would have reduced the set up requirement to 36.4 hours of funded support for minimal database/web programming. Assuming that 45% of free-text reports were exact duplicates of another report, we estimated that 70,891 medications would require *post hoc* coding if a free-text only data collection method was implemented. It is estimated that *post hoc* coding of medication reports would require 797.5 hours of support in this scenario. Thus, the overall time required to collect and code medications using free-text alone is estimated to be 833.9 hours, with an estimated cost of USD $0.20 per medication.

**Table 3:** Relative costing of AWARD framework versus traditional free-text only method.

	Unique text to code	Time (hours)^a^	Estimated cost (USD)^b^	Number of medication reports collected	Cost per medication report entry (USD)
**AWARD system**					
Database/web programming	-	73.5	$2279		
Set-up—curation of list of common medications	-	15.0	$465		
Set-up—coding of list of common medications	2025	25.3	$785		
Coding of free-text entries	2529	28.5	$882		
**TOTAL**	**4554**	**142.3**	**$4410**	**128,893**	**$0.03**
**Traditional free-text only approach**					
Database/web programming	-	36.4	$1127		
Coding of free-text entries	70891	797.5	$24,723		
**TOTAL**	**70891**	**833.9**	**$25,850**	**128,893**	**$0.20**

^a^Assumptions: 45 seconds per medication allocated for curation of list of common medications;

40.5 seconds allocated for coding of list of common medications (two coders—18.7 seconds each, plus 3.1 seconds per medication for discordance resolution given that 1 out of 6 medications were discordant and required coding by a third coder); 40.5 seconds allocated for coding of free-text entries (two coders plus a third coder for 1 out 6 medications); In the free-text only approach 45% of reports would be duplicate free text, hence only 55% of reported medications would require coding.

^b^Cost per hour calculated based on hourly rate of 31 USD for Research Officer and rounded to the next whole number.

## Discussion

We find that implementation of a two-pronged framework involving type-to-search functionality and free text entry dramatically reduced the time required to code medication data in a large-scale clinical trial. Common problems faced by medical coding experts, such as illegibility, spelling errors, use of abbreviations, local brands, and multiple medications recorded together [14,15], were avoided for 95.4% of all medication reports in ASPREE through the provision of the type-to-search strategy. The linkage of this box to the list of common misspellings allowed staff to self-correct transcription errors or misspelling and choose the correct medication from the type-to-search box at the time of data entry, contributing to the 6 fold reduction in cost of coding ASPREE’s structured medication data. However, if only the type-to-search box had been utilised key medications such as apixaban, would have been omitted from data collection. Providing the option for free-text entry produced a safety net and ensured all medications were collected. Two of the medications most commonly reported as structured data were also reported as free-text, indicating that staff felt comfortable utilising the free-text entry option if they were uncertain of the correct option to select from the type-to-search box.

The most common reason for free-text entry was that the appropriate option was not available in the type-to-search box. Given that 6 of the top 10 most commonly reported free-text medications were not available in the type-to-search box, *post hoc* coding could have been avoided for 20.3% of free-text reports if these six medications had been added to the reference lists and made available in the type-to-search box. Future studies wishing to implement this framework should be aware of this limitation and ensure that the options in the type-to-search box are kept up to date.

Training provides an opportunity for further optimisation of the medication data collection framework. While only a small proportion of ASPREE medications were collected as free-text, spelling errors and the provision of additional information were among the three most common reasons for entry as free-text. Almost all free-text reports of aspirin included additional information about dose, indicating that staff wanted to provide more information than was required based on the study protocol. Additional training about the intended use of the data and the rationale behind collection of such a limited concomitant medication dataset may have increased the proportion of medications entered via the type-to-search box and in turn reduced the time requirements for *post hoc* coding.

In research, there is always a compromise between limited resources and data quality [16–18]. While medication data does not reach the size and scale of ‘omics data, it is often unstructured data and thus requires significant handling prior to analysis. When medication data are collected for large scale clinical trials or population studies, the combination of the scale and structure of the data presents significant challenges. The traditional method of free-text medication data collection and *post hoc* coding by a clinician is a labour intensive processes that would have taken almost half a full working year (21, 38 hours weeks) to complete for ASPREE, which was not feasible. In the past, studies have restricted costs associated with concomitant medication collection by choosing not to collect detailed medication data, and instead collecting binary questions about specific medication classes of interest [19,20]. While this choice may be cost-effective, it may also limit the potential for analysis and discovery, given that medications of interest must be pre-specified prior to commencement of data collection. Implementation of a two-pronged framework for structured data collection offers a cost-effective alternative to the traditional free-text only method for collection of detailed concomitant medication data. Utilisation of the type-to-search box dramatically reduced the cost of medication collection and coding compared with the traditional method. The cost-effective process for collecting detailed, structured concomitant medication data presented here could be applied to any large-scale clinical trial or population study where concomitant medication use is an important covariate for analysis. Finally, the process here described could be applied to other, non-medication, data that is routinely collected as free text (such as clinical signs or symptoms). Although such an application of this process would need to be tailored to the individual setting and software, in general, utilising a type-to-search approach is likely to be cost effective and user friendly.

### Limitations

Others have noted the challenges of coding medication dose, particularly for medications where doses change frequently (*e*.*g*. warfarin) [16]. However, ASPREE’s protocol did not require collection of medication dose and therefore the process described herein is not optimised for the collection of dosage. Lack of data regarding dosage may limit future research utilising this coded medication data. Additionally, ASPREE did not record how many medications were initially entered with a misspelling but then corrected as a result of staff being offered the correct medication via the type-to-search box. Thus, detailed analysis of the utility of the linked common misspellings database tables is not possible. Furthermore, discussion of methods to improve the time required to manually code free-text medications, (*e*.*g*., natural language processing or other data science interventions) is beyond the scope of this paper. Further research into these areas would provide helpful guidance.

## Conclusion

Medicine is increasingly driven by pharmaceutical interventions and clinically relevant research must account for the role of concomitant medications. Traditional methods of medication collection and coding are prohibitively expensive and do not leverage modern digital infrastructures that provide an opportunity to collect comprehensive medication data from community participants in an efficient manner. Implementation of a two-pronged framework that includes a pre-configured type-to-search list of medications as an adjunct to free text data entry is a cost-effective alternative to collection of free-text data only. Ensuring options in the type-to-search list are kept up to date, and training staff will minimise the number of free-text entries. Higher initial set-up costs are justified by long-term cost effectiveness and additional potential for analysis and discovery gained through the collection of detailed medication data.

## Supporting information

S1 Table Reasons for entry of medications as free-text. (DOCX)

S2 Table Results of coding time trials. (DOCX)

S1 Fig Medication coder interface. (TIF)
