# Supplementary material for: Optimising medication data collection in a large-scale clinical trial
Source: PLoS One. 2019 Dec 27;14(12):e0226868. doi: 10.1371/journal.pone.0226868 (PMC6934269; doi:10.1371/journal.pone.0226868)
Supplement: S1 Table — (DOCX) [file pone.0226868.s001.docx]

**S1 Table: Reasons for entry of medications as free-text**

| **Reason** | **N** | **% of free-text** |
| --- | --- | --- |
| Not in type-to-search box | 2,041 | 44.6% |
| Spelling error  (e.g. Parindopril) | 865 | 18.9% |
| Additional information provided re dose, route or timing of medication  (e.g. Aspirin 81mg) | 626 | 13.7% |
| Trade name  (e.g. Lipitor) | 490 | 10.7% |
| Full chemical name  (e.g. Perindopril erbumine) | 283 | 6.2% |
| Both trade and generic name provided  (e.g. Cartia / Aspirin) | 122 | 2.7% |
| Combination with non-prescription medication  (e.g. Vitamin D and calcium) | 53 | 1.2% |
| Unclear why free text was entered  (i.e. free-text is exact match of option in type-to-search box) | 101 | 2.2% |
| TOTAL | 4581 | - |
