# Supplementary material for: Optimising medication data collection in a large-scale clinical trial
Source: PLoS One. 2019 Dec 27;14(12):e0226868. doi: 10.1371/journal.pone.0226868 (PMC6934269; doi:10.1371/journal.pone.0226868)
Supplement: S2 Table — (DOCX) [file pone.0226868.s002.docx]

**S2 Table: Results of coding time trials**

| **Detail** | **Result** |
| --- | --- |
| Number of coders completing time trial | 3 |
| Number of trials completed by each coder | 4 |
| Duration of each time trial | 15 mins |
| Minimum number of medications coded in any one time trial | 22 |
| Maximum number of medication coded in any one time trial | 79 |
| Total number of medications coded | 588 |
|  |  |
| Median number of seconds to code a medication (IQR) | 18.7 (22) |
