# Supplementary figures and images for: Optimising medication data collection in a large-scale clinical trial
Source: PLoS One. 2019 Dec 27;14(12):e0226868. doi: 10.1371/journal.pone.0226868 (PMC6934269; doi:10.1371/journal.pone.0226868)

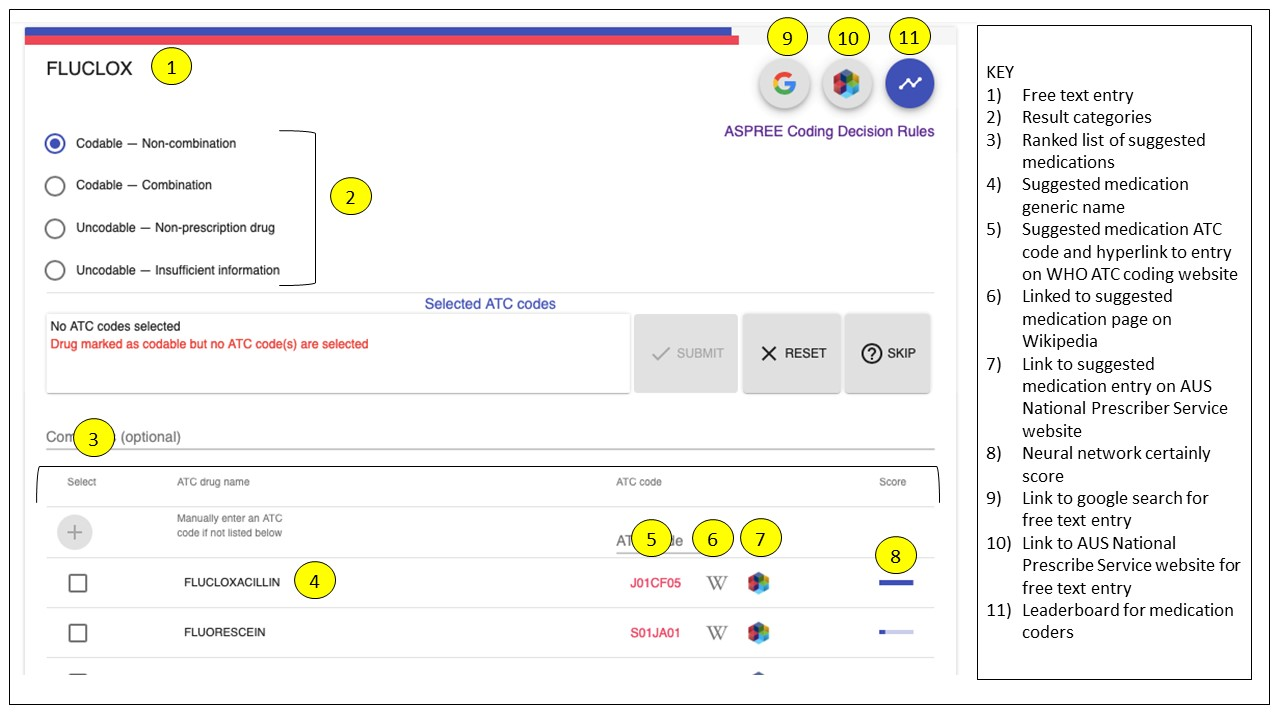

Supplement: S1 Fig — (TIF) [file pone.0226868.s003.tif]
